# Performance Evaluation on Profile Control and Flooding of QPD Thermosensitive Gel in High-Temperature and High-Salinity Reservoir of Block X

**DOI:** 10.3390/molecules31203643

**Published:** 2026-10-13

**Authors:** Fan Lu, Jihong Zhang, Xinjian Tan, Fei Li, Ziwei Qu, Qi Wang

**Affiliations:** 1 Key Laboratory of Enhanced Oil and Gas Recovery, Ministry of Education, Northeast Petroleum-University, Daqing 163318, China; lF187466179161@163.com (F.L.); klmytxj@163.com (X.T.); lifei110706@163.com (F.L.); 15046884715@163.com (Z.Q.);; 2 No. 8 Oil Production Plant, Daqing Oilfield Company, Daqing 163000, China

**Keywords:** high-temperature and high-salinity reservoir, partial quaternization, dimethyl carbonate (DMC), thermosensitive QPD gel, enhanced oil recovery

## Abstract

The reservoir of Block X is classified as a typical high-temperature and high-salinity formation, with a formation temperature of 83 °C and a total salinity of 16,000 mg/L. To further boost oil recovery, gel profile control and flooding was adopted to optimize the field development performance. Nevertheless, conventional phenolic gel systems suffer from poor gelling properties and insufficient stability under such harsh reservoir conditions, leading to unsatisfactory application outcomes. This creates an urgent demand for the development of an innovative gel system with superior adaptability. In this research, dimethyl carbonate, a green chemical reagent, was applied to partially quaternize poly(N,N-dimethylaminoethyl methacrylate) (PDMAEMA) copolymers, replacing traditional halogenated alkanes. This approach yielded a novel copolymer with enhanced thermal resistance. By blending the modified copolymer with stabilizers and crosslinking agents, a temperature-responsive gel system named Q-PDMAEMA-DMC (abbreviated as QPD gel) was successfully formulated. Multiple characterization methods were adopted to clarify the structural features and comprehensive performance of the QPD gel, including nuclear magnetic resonance spectroscopy, Fourier transform infrared spectroscopy, Zeta potential testing, SEM microtopography, gelling property evaluation, and rheological analysis. Core flooding tests were also conducted under laboratory conditions simulating the actual reservoir environment of Block X. The testing results reveal that the QPD gel maintains stable internal structure, reliable gelling performance and favorable rheological behavior across the temperature range of 50 °C to 100 °C. When deployed for profile control and flooding in Block X, the QPD gel delivered remarkable enhancement in oil recovery. Compared with conventional water flooding alone, the final recovery degree increased by 16.9 percentage points; meanwhile, it achieved an improvement of 6.2 percentage points relative to the original unmodified PDMAEMA gel system. These findings confirm that the QPD gel can effectively mobilize residual oil in high-temperature and high-salinity reservoirs. It therefore serves as a technically feasible and promising solution to further improve oil recovery efficiency in Block X.

## 1. Introduction

Conventional gel systems struggle severely with poor stability and inadequate thermal tolerance when deployed in harsh oil reservoirs featuring high temperature and high salinity [1]. As these gels migrate deep underground, thermal degradation and salting-out effects often weaken their network structure, sometimes even complete collapsing. This drastically undermines their plugging performance, greatly limiting the efficiency and practical application of deep profile control and flooding technologies [2,3]. Partially hydrolyzed polyacrylamide (HPAM) and its chromium/phenolic crosslinking systems, which are widely adopted in the industry, feature low cost. Even so, in extreme reservoir conditions above 80 °C and with salinity exceeding 10,000 mg/L, these materials suffer rapid backbone chain breakage and sharp viscosity loss. They also cannot actively respond to changes in reservoir physical properties [4,5]. For this reason, developing advanced polymer formulations with both environmental adaptability and intrinsic intelligent sensing capability has become a key research focus at the forefront of deep reservoir profile control.

As a typical thermoresponsive polymer, Poly(2-(dimethylamino)ethyl methacrylate) (PDMAEMA) has attracted extensive attention because of its unique lower critical solution temperature (LCST) behavior, which is generally tunable within the range of 35–45 °Cand varies with polymerization degree and solution concentration. In addition, PDMAEMA is a weakly alkaline polymer. Its protonation can be promoted in an acidic environment or by introducing CO_2_, whereas deprotonation occurs under alkaline conditions. Thanks to such multi-responsive properties, PDMAEMA and its derivatives exhibit great application potential in biomedical fields including gene delivery [6] drug delivery, and smart emulsions. To further enrich its functionalities, quaternization of the tertiary amine groups on PDMAEMA with alkyl halides has become a common modification strategy. Quaternized PDMAEMA homopolymers and copolymers have been widely applied in gas separation [7], antifouling and antibacterial materials [8,9], blood-compatible materials [10], adhesive materials [11], and other related areas. Traditional quaternization mostly relies on methyl iodide, as well as long-chain and short-chain alkyl halides as modification reagents. In related studies, Cheng and colleagues [12,13] adopted octadecyl iodide for quaternization, and regulated the quaternization degree to synthesize pH-responsive PDMAEMA molecular brushes. Their research proved that a low degree of quaternization could induce the formation of lipid bilayer-like structures. Another study by Vamvakaki et al. [14] focused on partially quaternized PDMAEMA diblock copolymers modified with methyl iodide. The team discovered that increasing the hydrophilicity of the copolymers would raise their LCST value or even eliminate the LCST effect entirely; meanwhile, slightly quaternized diblock copolymers exhibited excellent surface activity, delivering great potential for surfactant applications. Manouras et al. [15] implemented quaternization on PDMAEMA via chlorine substituted short and long-chain halogenated hydrocarbons, and reached a similar conclusion: quaternization alters the LCST of polymers and endows them with high surface activity. Traditional quaternization modifications heavily rely on highly toxic alkyl halide reagents, which pose considerable environmental and safety risks. Additionally, it remains challenging to achieve precise quantitative control over the quaternization degree, residual tertiary amine ratio, and the synergistic mechanism of multiple response properties [16].

To address the above limitations, this study innovatively adopts dimethyl carbonate (DMC), a green, low-toxic and biodegradable agent, as the quaternizing reagent to carry out controlled partial quaternization modification on PDMAEMA. A novel thermo-responsive gel system (QPD) was thereby successfully constructed. This new formulation completely eliminates the reliance on toxic modifiers. By precisely tuning the degree of quaternization and the proportion of residual tertiary amine groups, it achieves a synergistic improvement in structural stability, thermal tolerance and deep reservoir plugging performance. The research offers a promising new strategy for enhanced oil recovery in high-temperature and high-salinity reservoirs.

## 2. Results and Discussion

### 2.1. Structural Characterization and Verification

#### 2.1.1. Proton Nuclear Magnetic Resonance (^1^H NMR) Analysis

**Figure 1:**
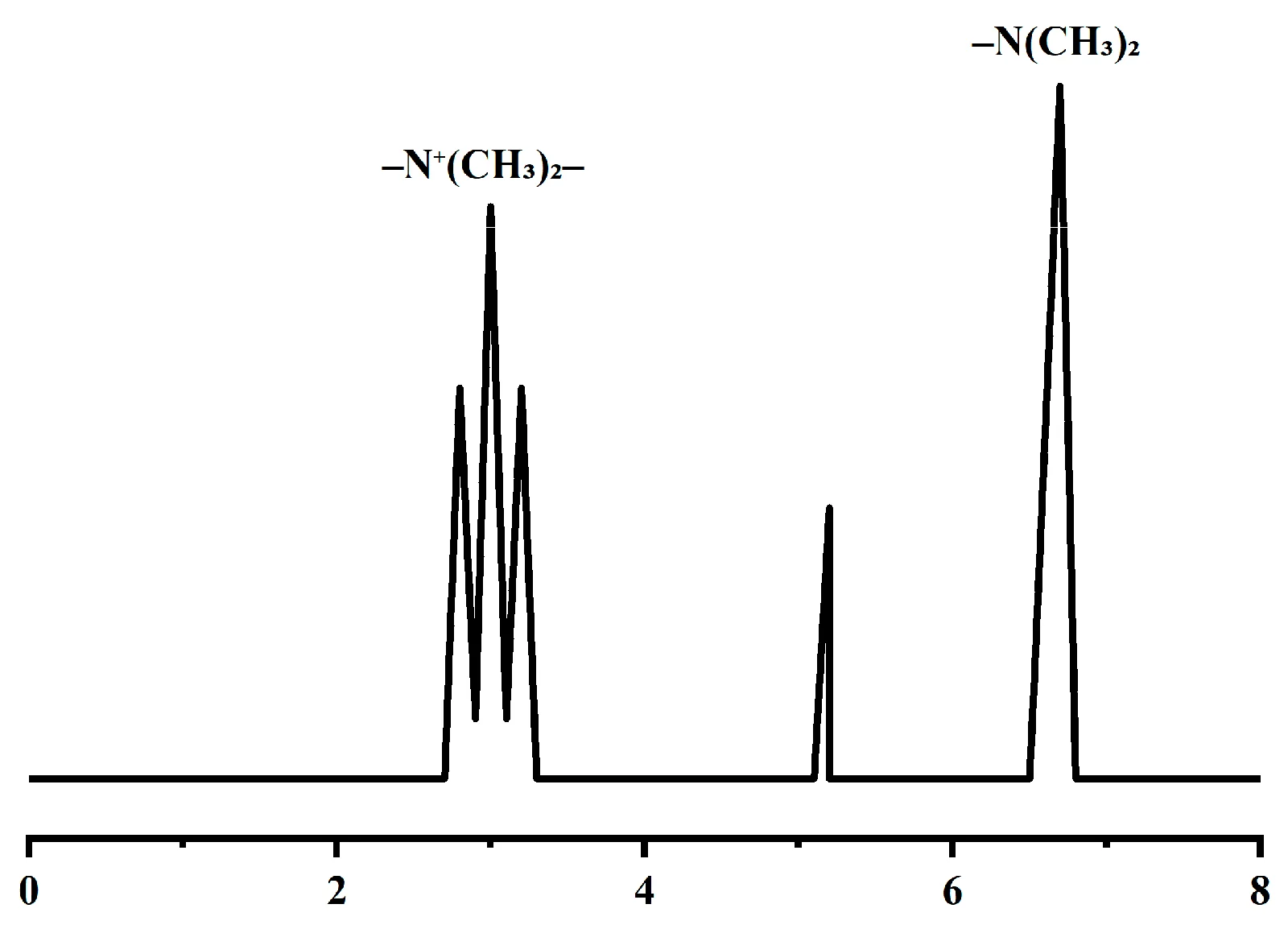
^1^H NMR spectrum of QPD.

The ^1^H NMR spectrum of QPD is shown in Figure 1. A strong and broad triplet observed at δ = 3.2–3.4 ppm corresponds to the methyl protons of the quaternary ammonium group –N^+^(CH_3_)_2_–. Meanwhile, the signal intensity assigned to the methyl protons of residual tertiary amine –N(CH_3_)_2_ remains relatively weak within the range of δ ≈ 2.2–2.4 ppm. This finding confirms that a considerable portion of tertiary amine groups has been successfully converted into quaternary ammonium structures.

#### 2.1.2. Fourier Transform Infrared Spectroscopy (FTIR) Analysis

**Figure 2:**
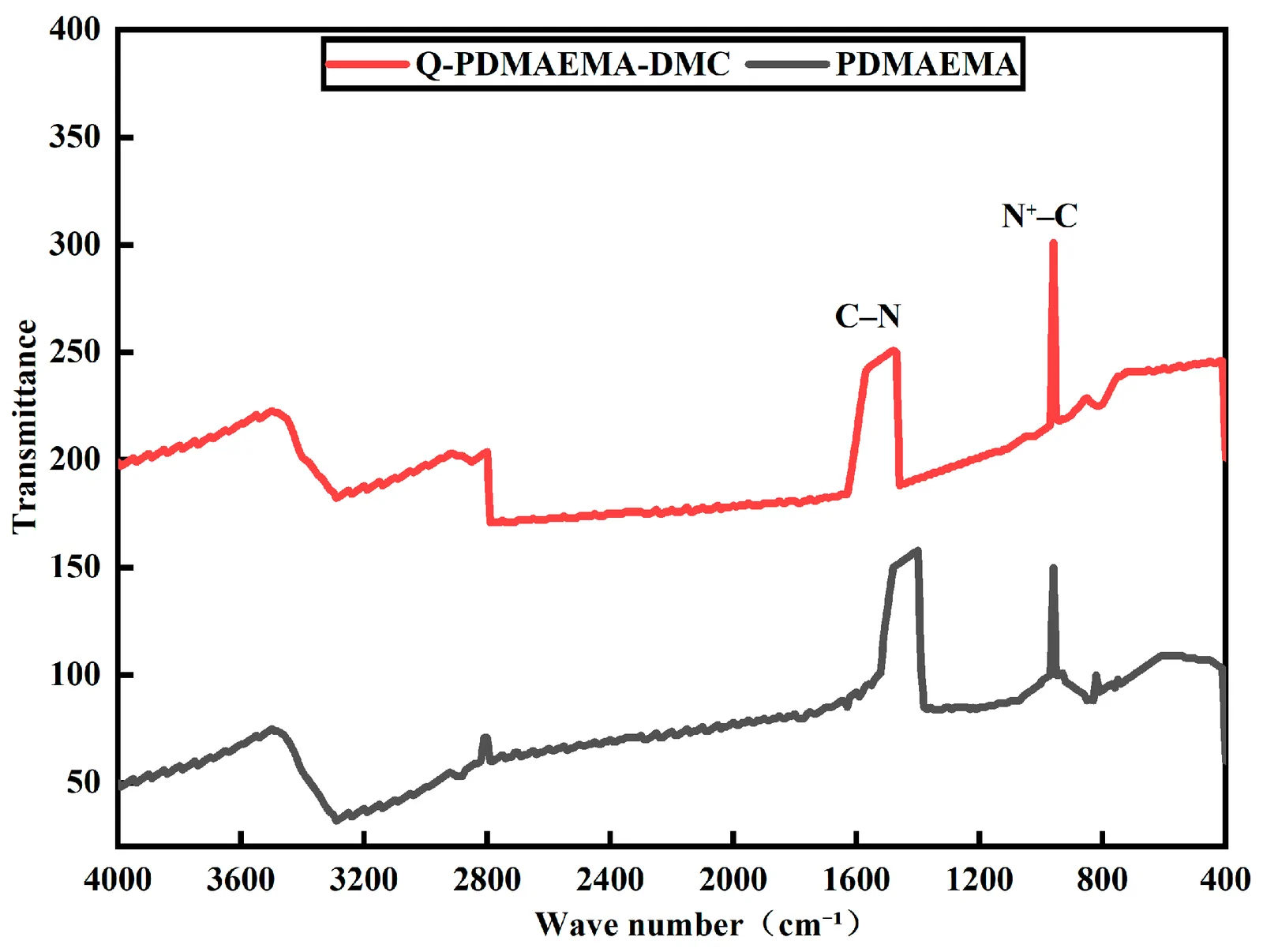
FTIR spectra of PDMAEMA and QPD.

The FTIR spectra of PDMAEMA and QPD are shown in Figure 2. FTIR characterization reveals that Q-PDMAEMA-DMC exhibits a moderate absorption peak assigned to the C-N stretching vibration at 1480 cm^−1^. Compared with pure PDMAEMA, the N^+^-C skeletal vibration signal at 960 cm^−1^ is markedly intensified in the modified sample. These spectral changes clearly demonstrate that tertiary amine moieties have been effectively converted into quaternary ammonium groups, which further validates the intact chemical structure of the target product.

#### 2.1.3. Micromorphology Analysis

**Figure 3:**
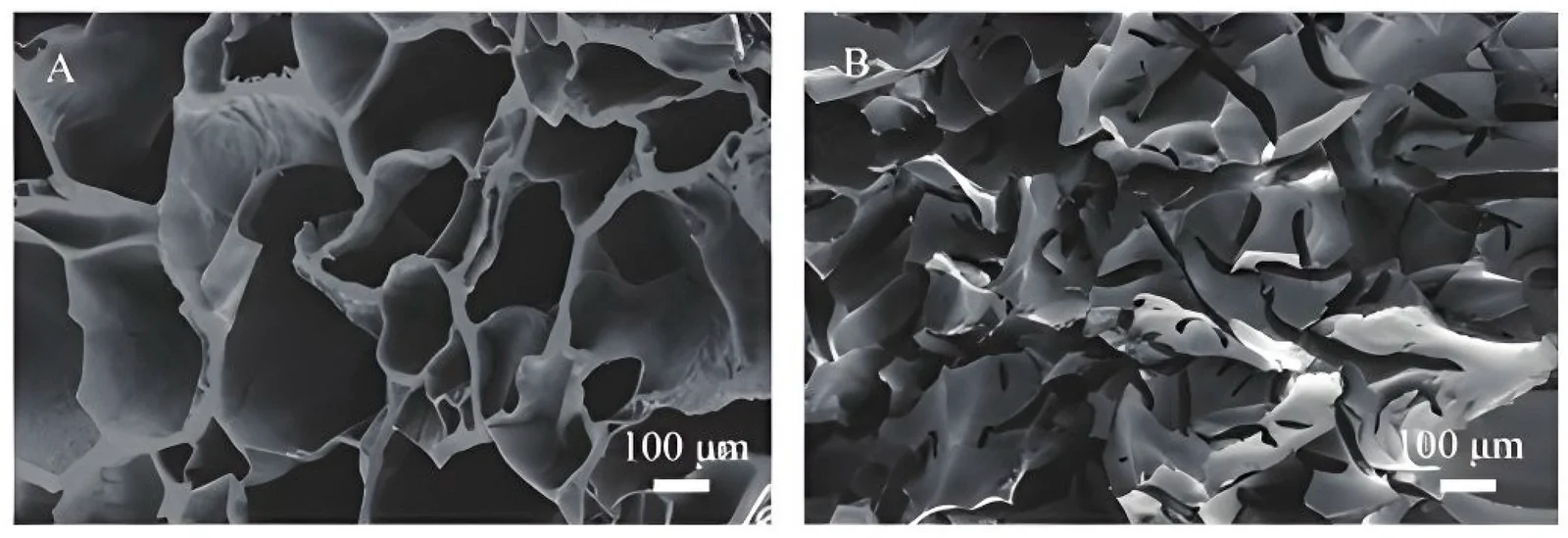
Micromorphology of freeze-dried gel samples: (**A**) PDMAEMA, displaying a loose macroporous three-dimensional network with large pores, thin pore walls, and good interconnectivity; (**B**) QPD, exhibiting a denser and more stable network structure with fewer macropores, reduced pore size, and thicker pore walls.

The changes in the microstructure of the gel system before and after modification were characterized by scanning electron microscopy, and the results are shown in Figure 3. As can be seen from Figure 3A, the unmodified PDMAEMA gel exhibited a microstructure characterized by large pores, thin pore walls, and a relatively smooth surface after freeze-drying. This is because the molecular chains of the unmodified PDMAEMA gel contained only tertiary amine groups, lacking strong cationic electrostatic repulsion. The physical crosslinking between molecular chains relied mainly on weak hydrophobic association and hydrogen bonding, resulting in a low crosslinking density and an insufficiently dense network skeleton. Consequently, a loose, macroporous three-dimensional network with good interconnectivity was formed during the freeze-drying process. As shown in Figure 3B, the microstructure of the modified QPD gel underwent significant changes. Compared with the PDMAEMA gel, the number of macropores per unit area in the QPD gel was markedly reduced, the pore size decreased, the pore walls thickened, and the overall structure became much denser. This is attributable to the introduction of quaternary ammonium groups (–N^+^(CH_3_)_2_–), which enhanced the electrostatic interactions and hydrogen bonding between molecular chains, thereby rendering the network structure more stable.

#### 2.1.4. Zeta Potential Analysis

**Figure 4:**
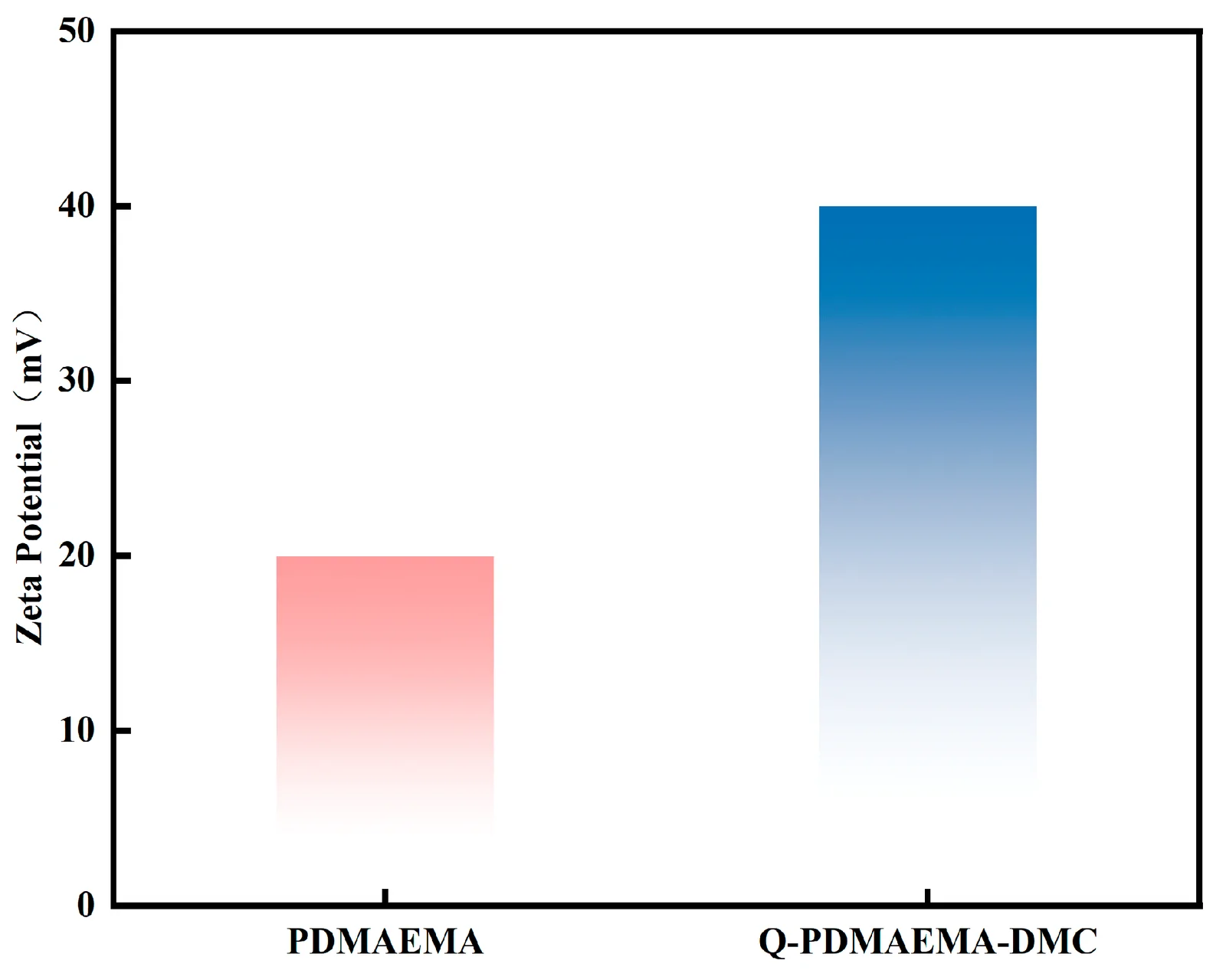
Zeta potential of PDMAEMA and QPD.

Zeta potential is a critical parameter for characterizing the surface charge properties of colloidal systems, and its measurement principle is rooted in Stern’s electrical double layer theory [17]. When charged particles undergo electrophoresis under an applied electric field, oppositely charged ions in the dispersion medium are attracted to the particle surface. Under strong electrostatic attraction, some ions are tightly bound around the particle, forming the Stern layer (compact layer). Ions farther from the particle surface experience weaker electrostatic interactions and form a loosely structured diffuse layer. Between the Stern layer and the diffuse layer lies a hypothetical boundary—the slipping plane. When the particle moves under the electric field, ions within this plane migrate with the particle, while those outside remain stationary. The electrical potential at the slipping plane relative to the bulk liquid is defined as the Zeta potential, also known as the electrokinetic potential [18]. Accordingly, the Zeta potentials measured for the PDMAEMA and QPD gel systems are illustrated in Figure 4. Experimental data reveal that the Zeta potential of QPD rises markedly from the initial +20 mV of pure PDMAEMA to +40 mV. This clearly indicates that the strongly positively charged -N+(CH3)_2_ groups introduced during quaternization effectively enhance the overall charge density. The obvious increase in Zeta potential confirms successful exposure of quaternary ammonium groups on the colloid surface. Through electrostatic repulsion, these surface charges further drive the molecular chains to adopt an extended conformational structure. These results are consistent with previous studies on quaternized polymer systems. Vamvakaki et al. reported that partial quaternization of PDMAEMA diblock copolymers significantly increased their surface charge and altered their solution properties. Similarly, Manouras et al. emonstrated that quaternization of PDMAEMA using halogenated hydrocarbons led to a notable increase in Zeta potential, which enhanced the polymer’s amphiphilic behavior. The Zeta potential increase of +20 mV observed in our study is comparable to the trends reported in these works, further validating the effectiveness of the DMC-mediated quaternization strategy. Notably, while achieving a similar level of surface charge enhancement, our approach offers a greener alternative by using DMC instead of conventional toxic alkyl halides.

### 2.2. Gelation Performance Evaluation

**Figure 5:**
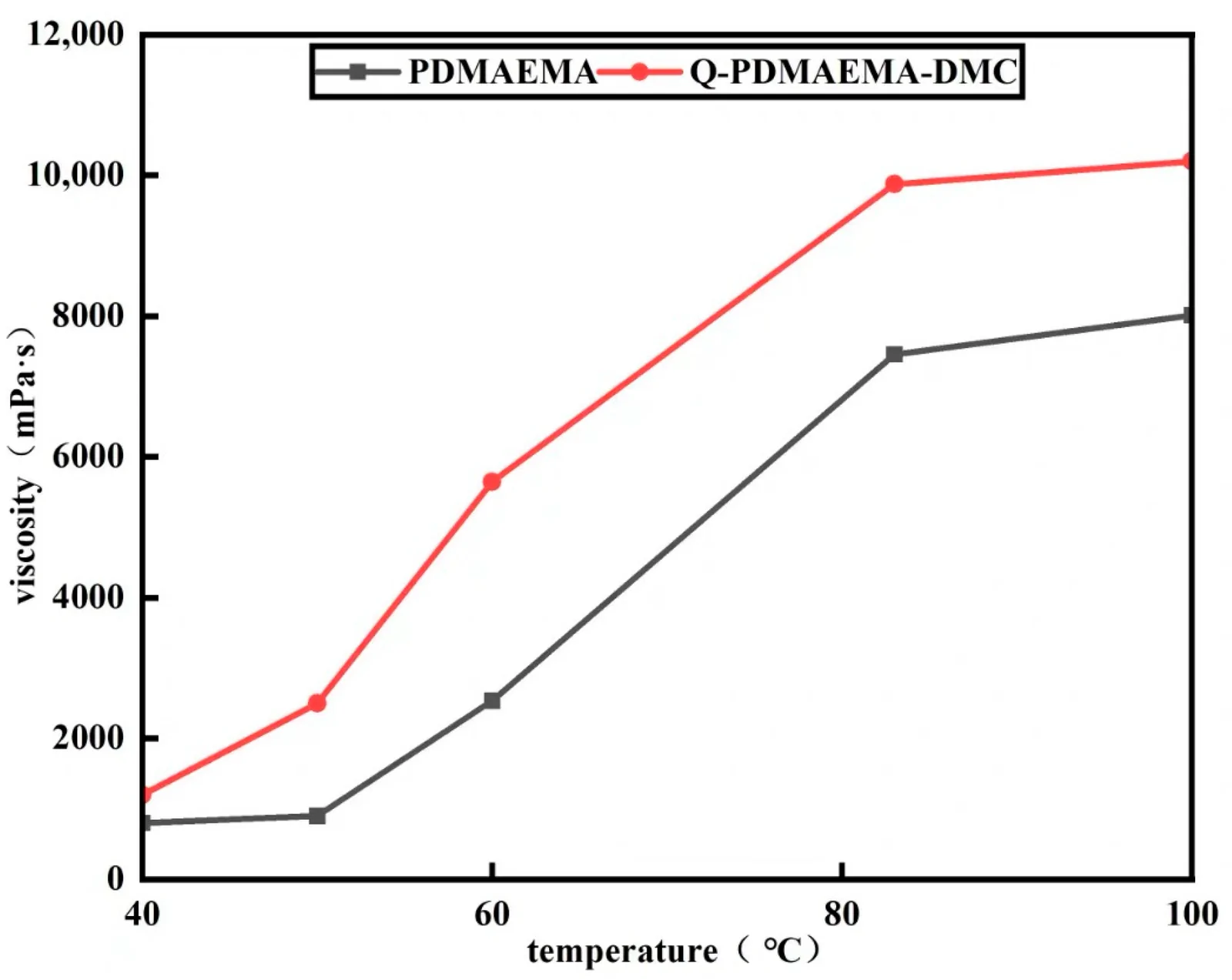
Viscosity variation curves of PDMAEMA and QPD systems with temperature.

**Figure 6:**
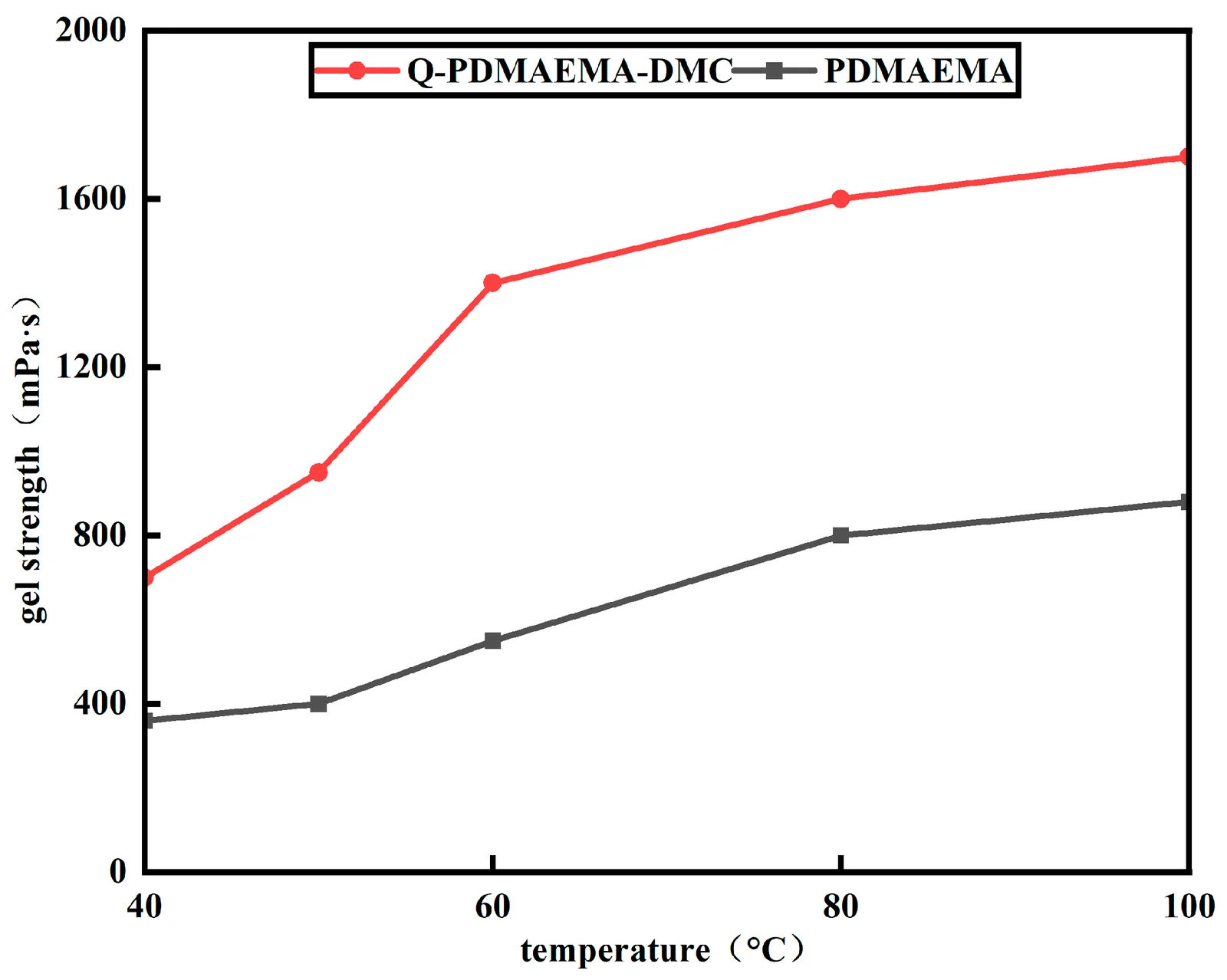
Curves of temperature versus gel strength for PDMAEMA and QPD systems.

Figure 5 and Figure 6 illustrate how PDMAEMA and QPD differ in their gelling characteristics. Across the full temperature range tested, the QPD system consistently maintained noticeably higher viscosity than the unmodified PDMAEMA material. This clearly shows that introducing quaternary ammonium groups and DMC monomers can effectively optimize overall rheological performance. With rising temperature, viscosity increased for both formulations, yet the QPD system displayed a much faster growth rate and stronger thermal thickening response. This enables it to naturally build greater flow resistance under high formation temperatures, making it highly effective for plugging high-permeability reservoir zones.

At the same temperature conditions, QPD formed far stronger gel structures than PDMAEMA, while reaching the targeted gel strength at a lower critical temperature. Under harsh high-temperature and high-salinity environments, conventional PDMAEMA molecular chains tend to expand or dissociate, preventing the formation of robust crosslinked networks. In contrast, the permanently positively charged chains of QPD promote physical crosslinking cores through hydrophobic association at elevated temperatures. Meanwhile, electrostatic repulsion along the polymer backbone stretches the molecular chains outward, creating a tightly packed network with dense internal structures and hydrophobic outer regions. This special structural feature effectively restrains thermal shrinkage, greatly improving the gel’s compactness, thermal stability, and salt tolerance under reservoir conditions.

### 2.3. Rheological Performance Analysis

**Figure 7:**
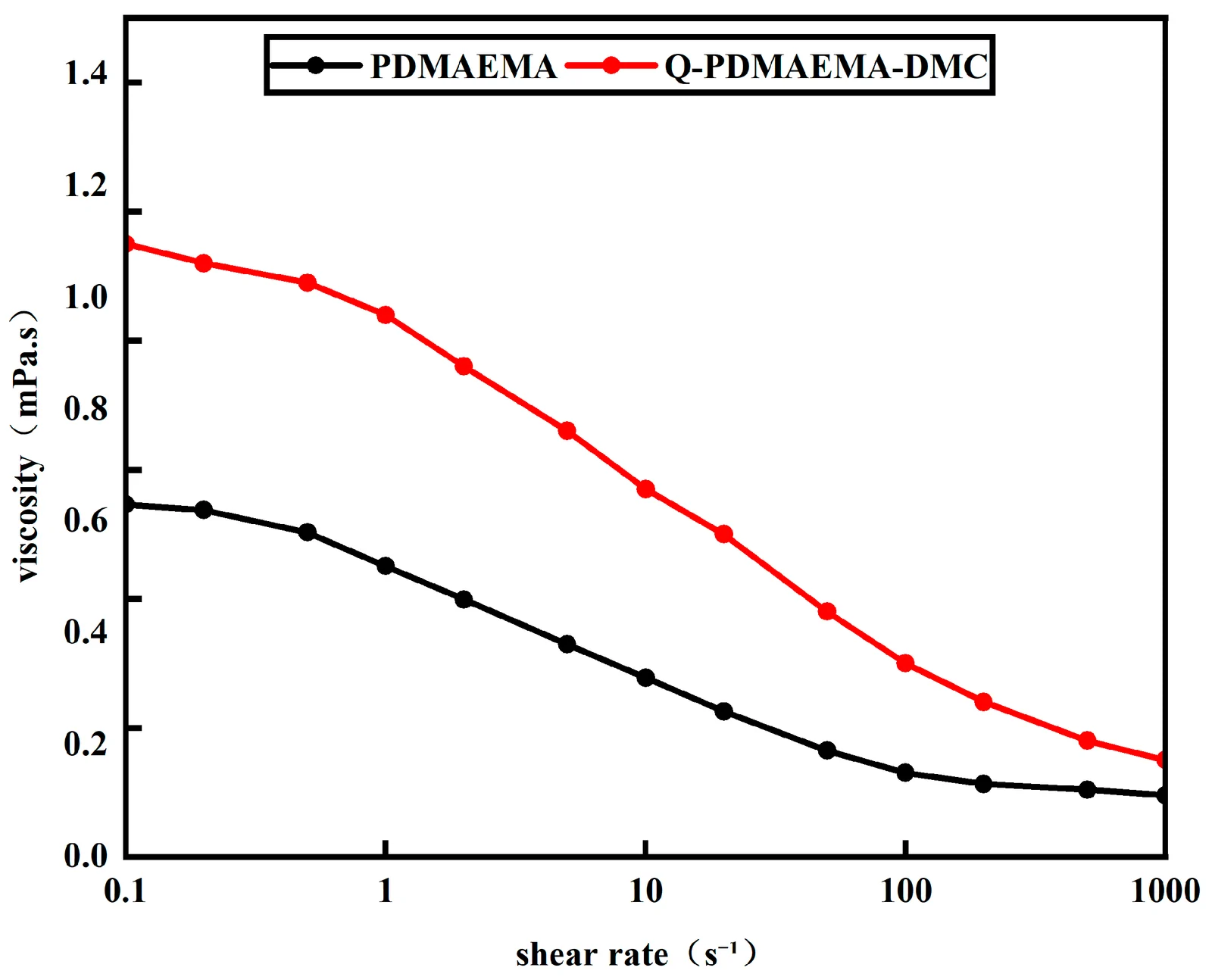
Comparison of steady-state shear rheological properties between PDMAEMA and QPD.

The rheological characteristic of a pseudoplastic fluid is that when the shear rate increases from zero—i.e., when the fluid flows very slowly—the shear viscosity remains essentially constant. As the shear rate continues to increase, the shear viscosity decreases with increasing shear rate, eventually approaching a fixed value [19]. As shown in Figure 7, the steady shear rheological curves of PDMAEMA and QPD are presented on a logarithmic scale. Both systems exhibited typical shear-thinning behavior consistent with pseudoplastic fluid characteristics. The zero-shear viscosity of QPD was significantly higher than that of PDMAEMA. This result indicates that quaternization modification introduces electrostatic repulsion, which transforms the QPD molecular chains from a coiled to an extended conformation, increases the hydrodynamic volume, and densifies the physical crosslinking network, thereby substantially enhancing the zero-shear viscosity. Meanwhile, at low shear rates, the viscosity of the QPD gel system decreased sharply, with a pronounced downward trend in the curve. This suggests the presence of extensive molecular chain entanglements within the system; once shear stress is applied, the molecular chains rapidly undergo relatively complete disentanglement, leading to a significant reduction in viscosity at low shear rates. This rheological characteristic enables the system to achieve deep reservoir plugging while maintaining good injectability during oilfield injection operations.

**Figure 8:**
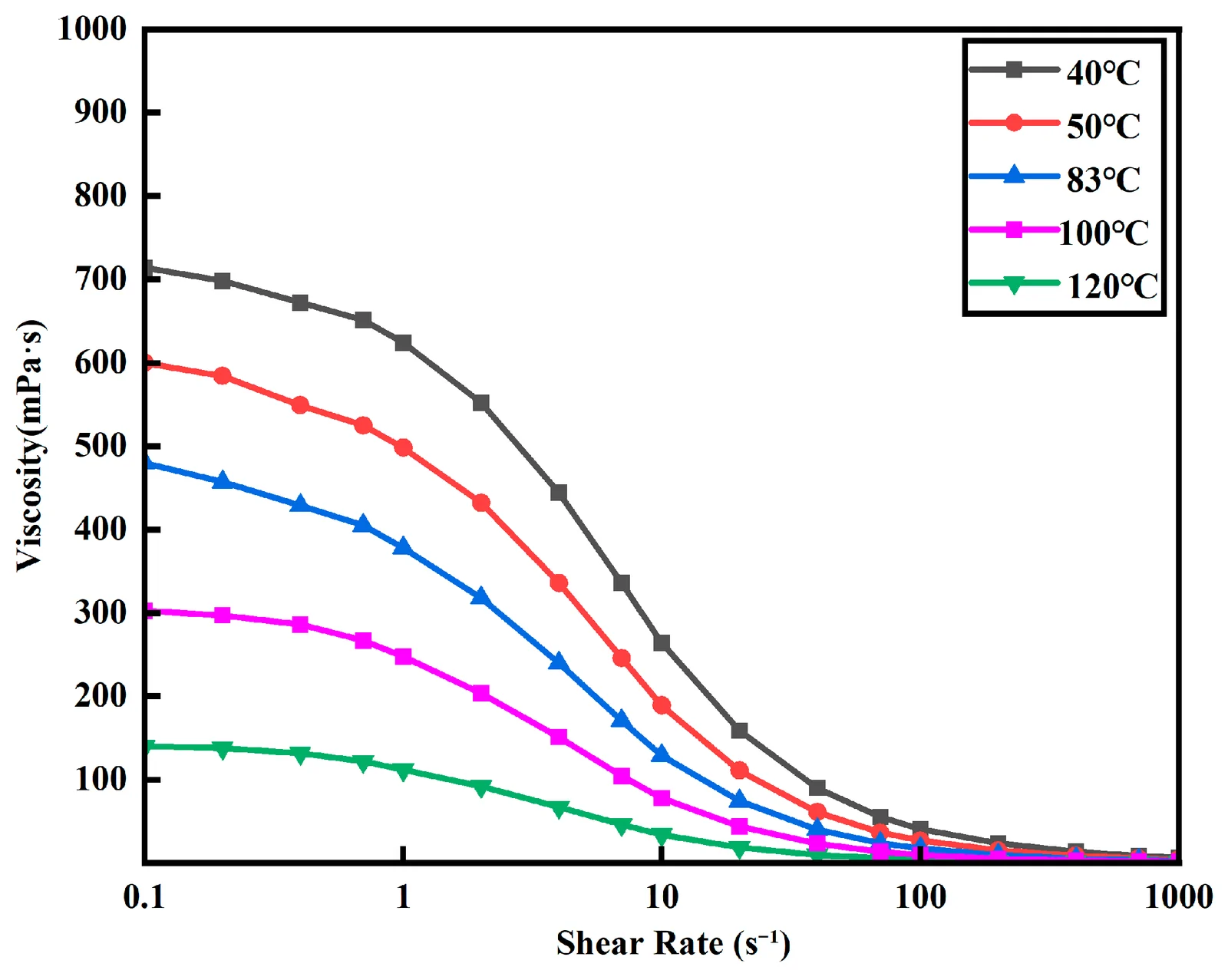
Rheological test results of PDMAEMA at different temperatures.

**Figure 9:**
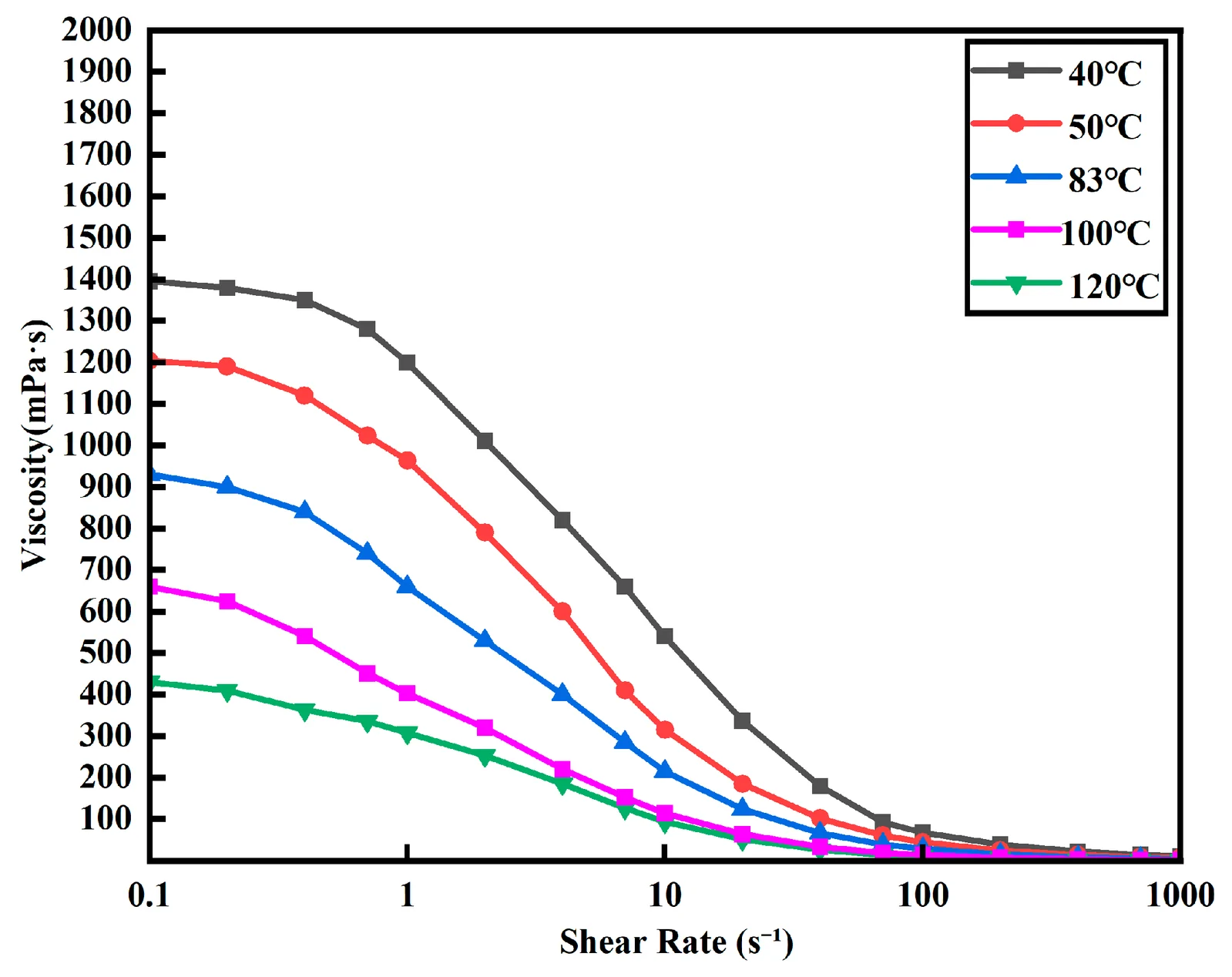
Rheological test results of QPD at different temperatures.

The steady shear rheological curves of PDMAEMA and QPD at different temperatures are shown in Figure 8 and Figure 9 on a logarithmic scale (*x*-axis). Both materials exhibit typical shear-thinning characteristics; however, QPD demonstrates significantly higher viscosity and superior thermal stability compared with PDMAEMA. At low shear rates, PDMAEMA relies primarily on non-covalent physical crosslinking, such as hydrogen bonding and hydrophobic interactions, to maintain its network structure. As temperature increases, intensified thermal motion rapidly weakens these non-covalent interactions, accelerating network disintegration. The zero-shear viscosity of PDMAEMA is 700 mPa·s at 40 °C and gradually decreases with rising temperature, accompanied by a shortened Newtonian plateau at low shear rates, indicating that its internal structure is highly unstable under elevated temperatures. In contrast, QPD, owing to the introduction of quaternary ammonium groups (–N^+^(CH_3_)_3_), possesses strong electrostatic repulsion and enhanced molecular chain rigidity, enabling it to maintain a relatively high viscosity even under high-temperature (120 °C) and high-shear conditions. This behavior confirms the favorable thermal stability of its network skeleton. Such a pronounced temperature–shear synergistic response fully demonstrates that quaternization modification effectively enhances the structural strength and deformation resistance of the gel network.

**Figure 10:**
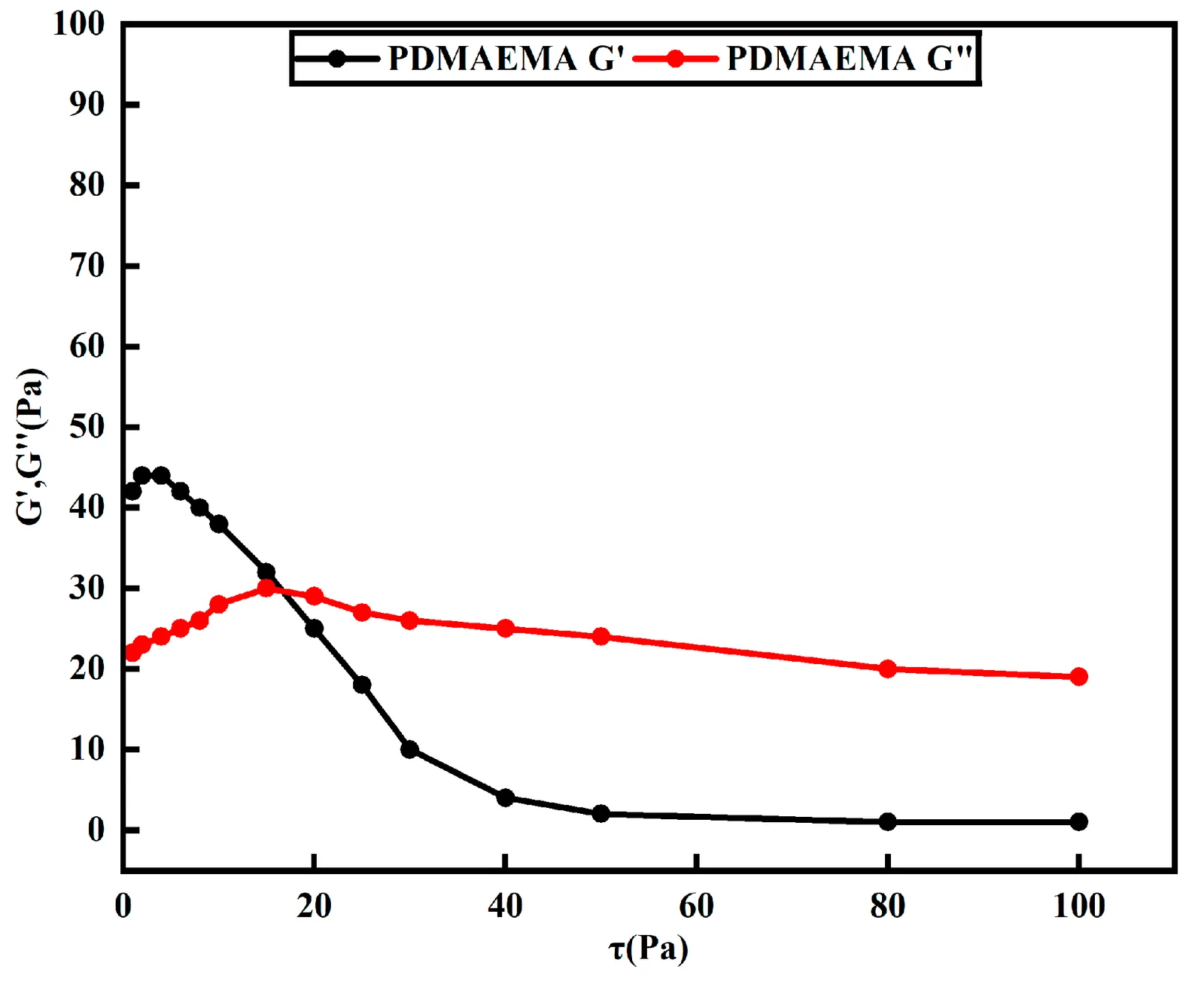
Stress sweep curve of PDMAEMA.

**Figure 11:**
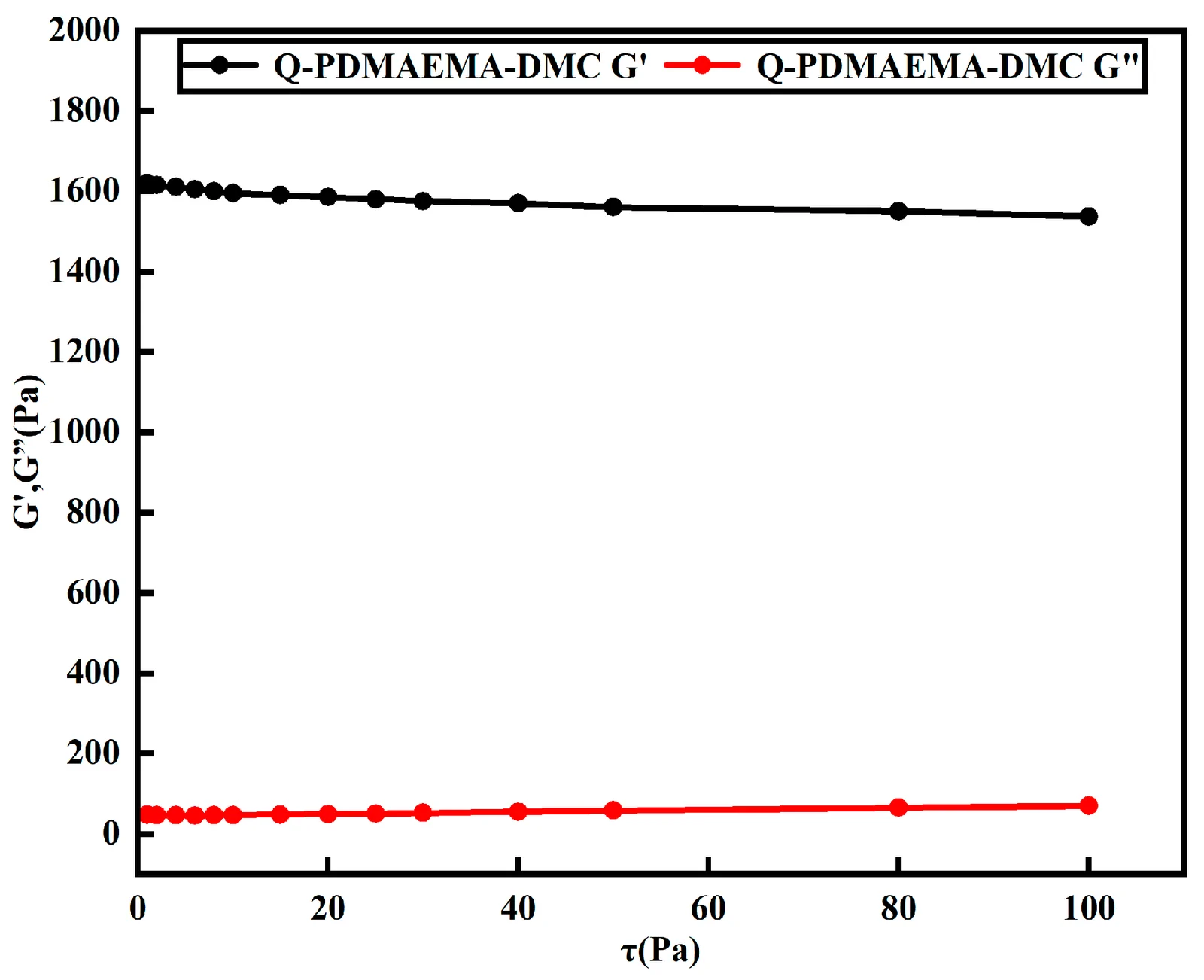
Stress sweep curve of QPD.

The variations in storage modulus (G′) and loss modulus (G″) of PDMAEMA and QPD gel systems with shear stress (τ) are shown in Figure 10 and Figure 11. The linear viscoelastic region (LVR) was determined through stress sweep tests, based on which an appropriate stress value was selected for subsequent frequency sweep tests to analyze the viscoelastic changes in the system before and after modification. The LVR reflects the structural strength of the system. As shown in Figure 11, the modified QPD gel system exhibits remarkably high structural strength, with a significantly broader LVR than that of the unmodified PDMAEMA solution. When the stress reaches 100 Pa, the storage modulus (G′) of the QPD gel remains stable with no obvious decline, demonstrating a wide linear response range. In contrast, the LVR of the PDMAEMA solution is relatively narrow; when the stress exceeds 5.0 Pa, its storage modulus (G′) drops sharply, indicating a weaker internal structure susceptible to mechanical disruption. This phenomenon is attributed to the introduction of stronger intermolecular positive–negative charge interactions in the modified QPD gel system, which constructs a more stable and denser three-dimensional network than that of the PDMAEMA solution. Considering the linear response ranges of both systems and to ensure that both QPD and PDMAEMA remain within the intact LVR, a fixed stress of 5 Pa was ultimately selected for subsequent frequency sweep tests.

**Figure 12:**
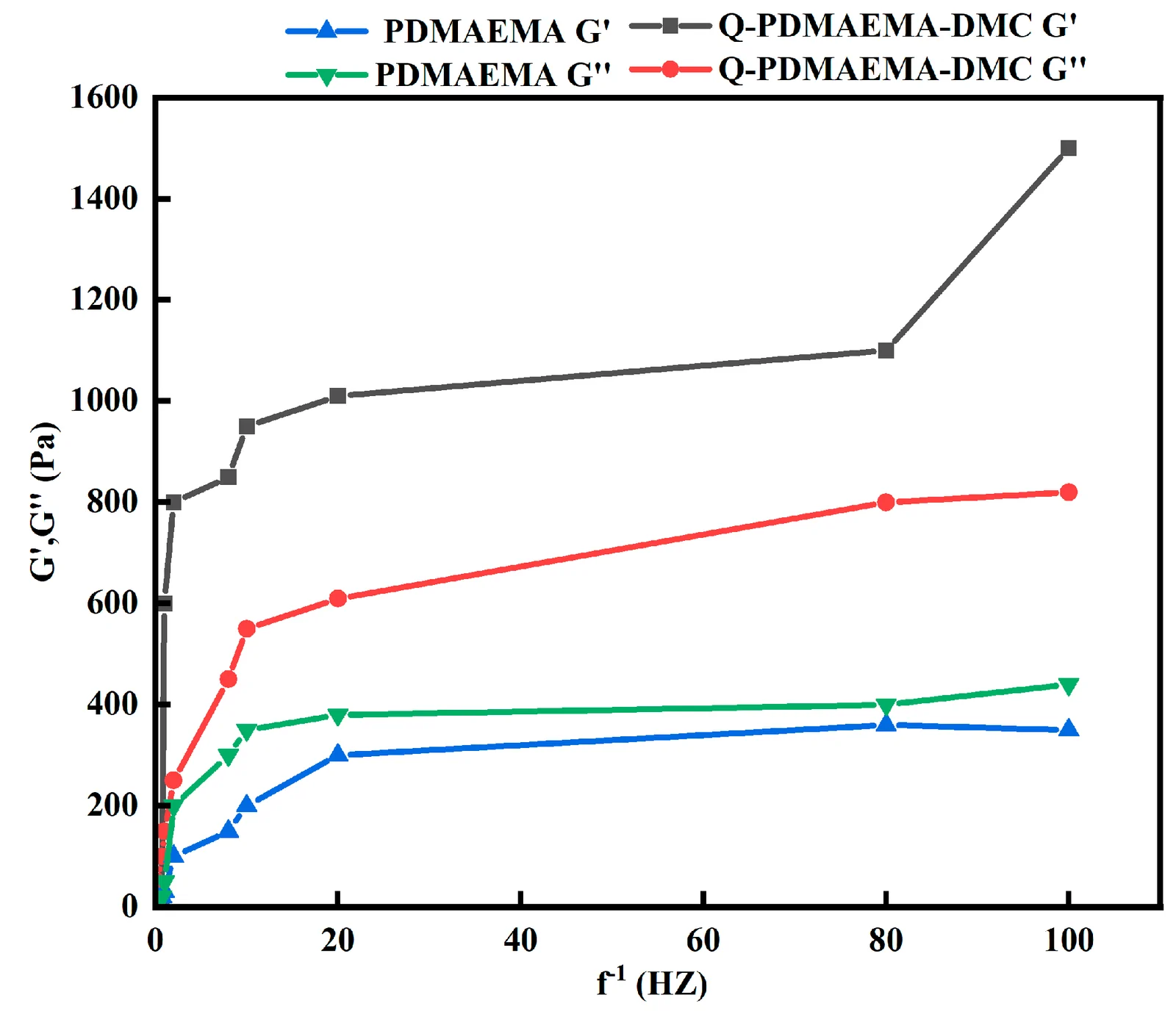
Frequency sweep curves for storage modulus.

**Figure 13:**
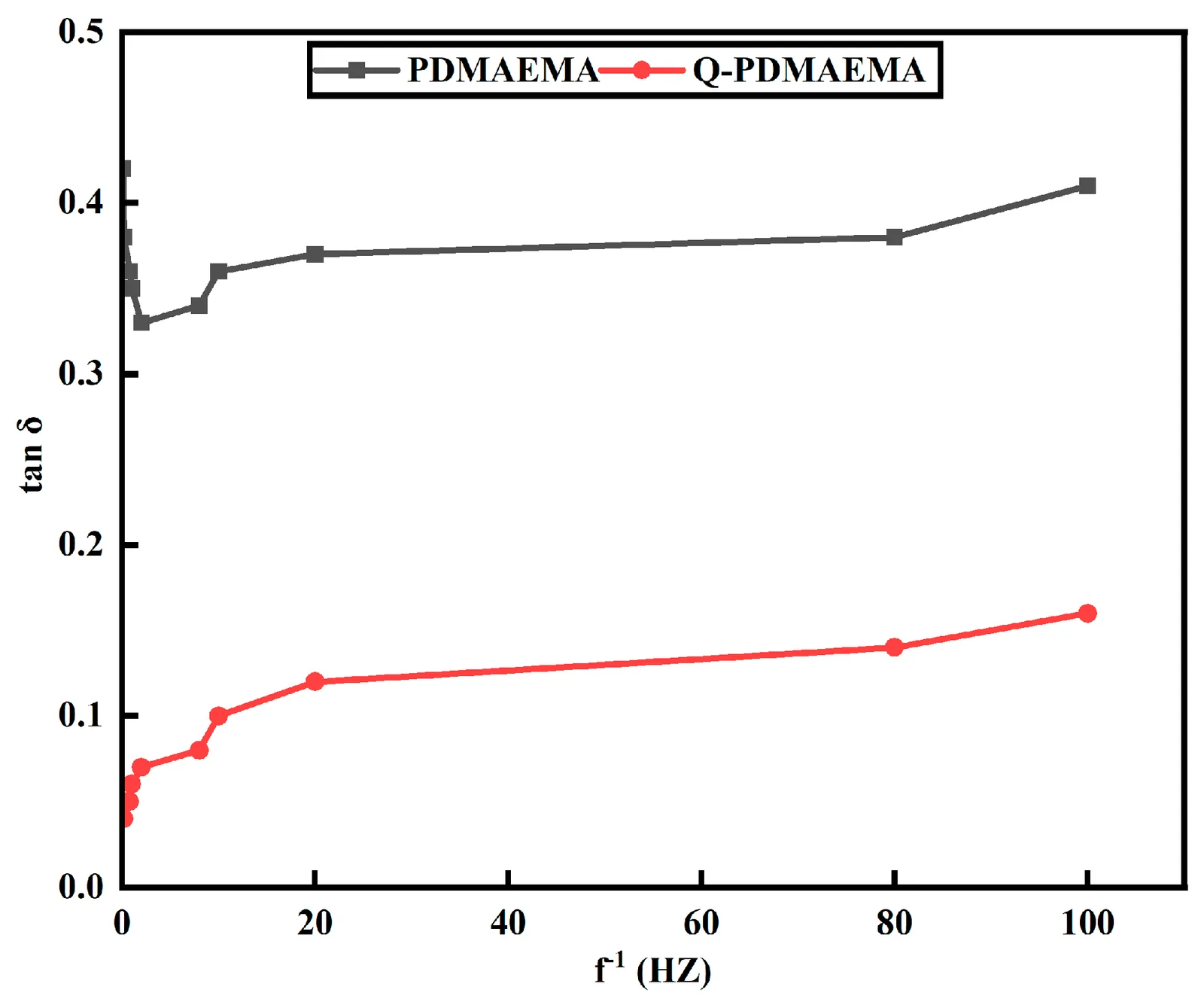
Dynamic frequency sweep curves.

The viscoelastic properties of the gel are characterized by the variations in the storage modulus (G′) and loss modulus (G″) as functions of oscillation frequency. The storage modulus (G′) represents the material’s ability to store elastic deformation energy, reflecting its capacity to recover after deformation and thus indicating solid-like behavior. The loss modulus (G″) represents the material’s viscous behavior, measuring the energy dissipated as heat during deformation when the applied stress is removed, thereby reflecting liquid-like behavior [20]. Based on the above, dynamic frequency sweep tests were conducted to evaluate the viscoelastic characteristics of both gel systems. As shown in Figure 12 and Figure 13, when measured at 25 °C under a fixed stress of 5 Pa and a concentration of 200 mmol/L, the QPD gel exhibited a markedly higher storage modulus (G′) than loss modulus (G″) across the tested frequency range. Its low tan δ value (G″/G′) reflected typical solid-like characteristics and outstanding elastic stability. In contrast, pure PDMAEMA only displayed weak gel performance under the same conditions. This demonstrates that quaternary modification, through enhanced electrostatic repulsion, constructs a densely crosslinked network with significantly improved deformation resistance and structural recovery capability.

### 2.4. Oil Flooding Performance Evaluation

**Figure 14:**
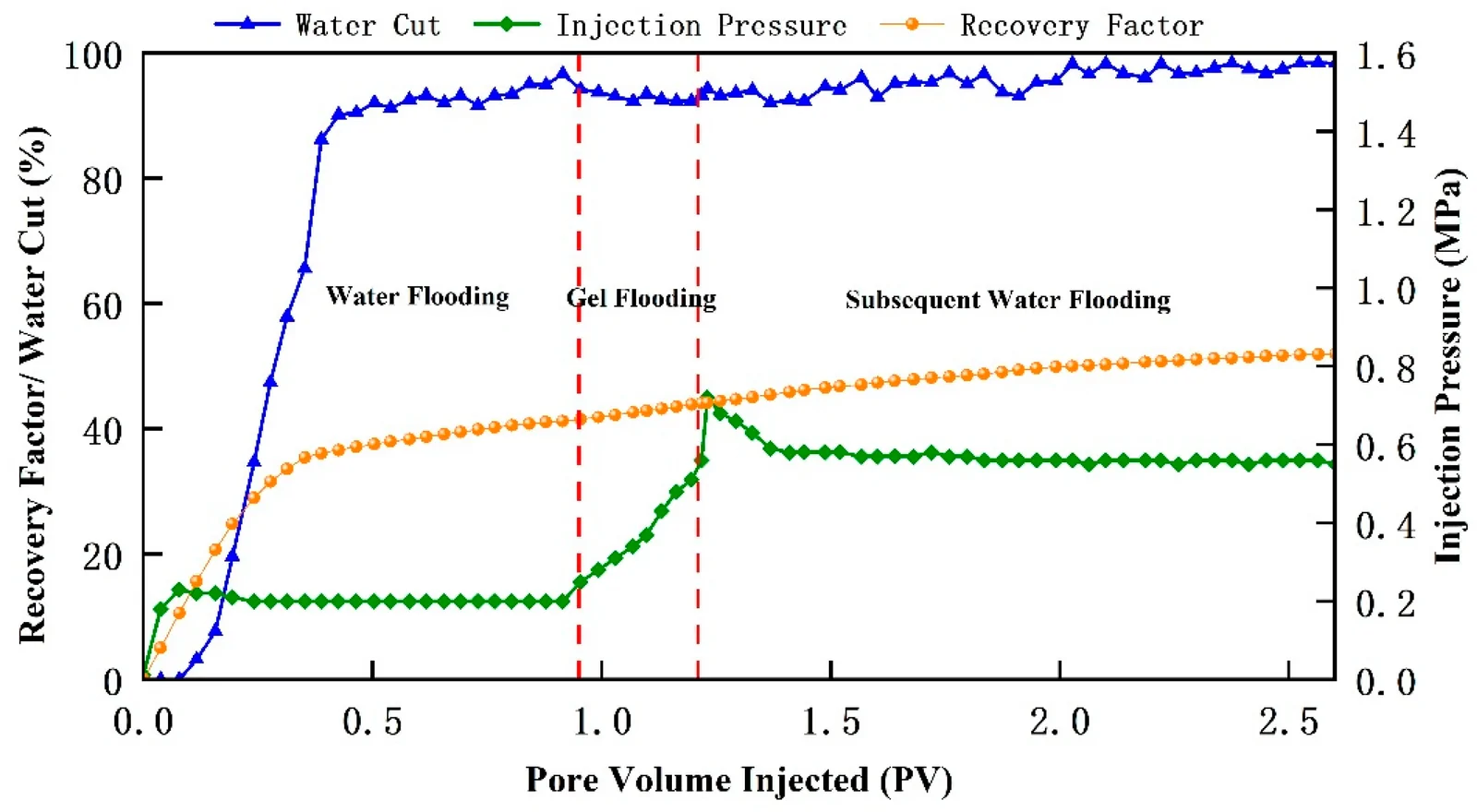
Oil flooding experiment of PDMAEMA microgel system. (The red dotted line serves as a visual guide to highlight the trend of the injection pressure curve).

**Figure 15:**
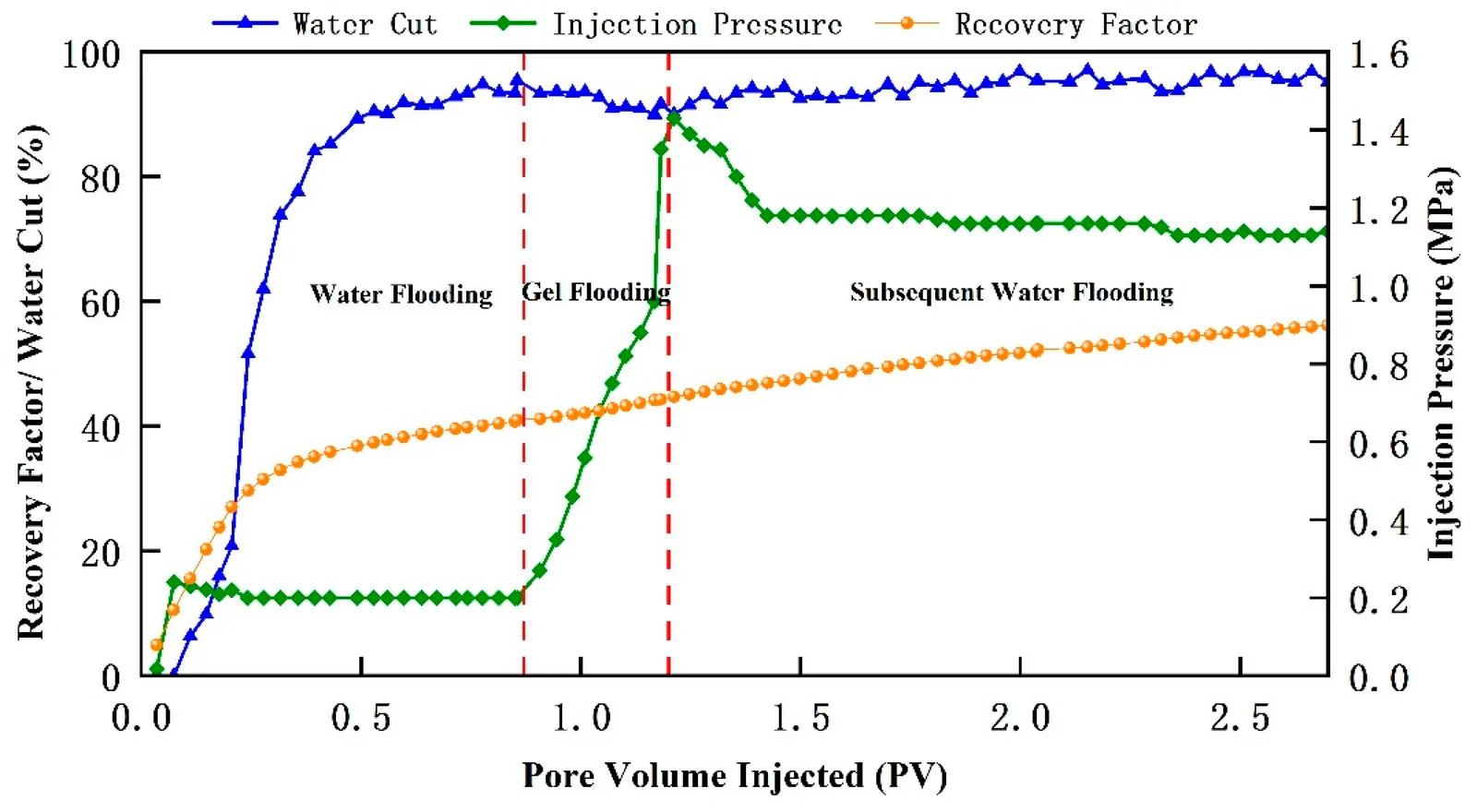
Oil flooding experiment of QPD microgel system. (The red dotted line serves as a visual guide to highlight the trend of the injection pressure curve).

Core flooding test results shown in Figure 14 and Figure 15 reveal that under high-temperature and high-salinity conditions of 83 °C and 16,000 mg/L, the injection pressure of the QPD gel system reached 1.4 MPa, which was obviously higher than that of the PDMAEMA gel system. After gel formation, QPD delivered strong plugging performance and favorable deep migration capacity, enabling effective profile control and fluid diversion within the core. In contrast, the PDMAEMA system produces a far lower injection pressure. It fails to develop sufficient gel strength in high-temperature, high-salinity environments, making deep reservoir plugging unfeasible; injected fluids consequently tend to breakthrough rapidly along dominant flow channels. In the subsequent water flooding stage, the water cut decreased significantly with the QPD treatment. The final oil recovery reached 60%, reflecting an improvement of 16.9 percentage points compared with conventional water flooding, and a 6.2 percentage point increase over the PDMAEMA system. Limited by inadequate thermal stability and salt resistance, the PDMAEMA group only achieved a final recovery rate of 55%, an enhancement of just 10.7 percentage points relative to baseline water flooding alone.

## 3. Materials and Methods

### 3.1. Experimental Materials and Instruments

2-(Dimethylamino)ethyl methacrylate (DMAEMA), Shanghai Macklin Reagent Co., Ltd., Shanghai, China; 2-(Dodecylthiocarbonothioylthio)-2-methylpropanoic acid (DMP), Azobisisobutyronitrile (AIBN), Dimethyl carbonate (DMC), Macklin Reagent Co., Ltd., Shanghai, China; BS-99 stabilizer, YFJJ alkaline crosslinking agent, 1,4-Dioxane (DO), heavy water (D_2_O), anhydrous methanol, deionized water, Sinopharm Chemical Reagent Co., Ltd., Shanghai, China.

50 mL stainless steel high-pressure reactor (Parr Model 4848 series, Parr Instrument Company, Moline, IL, USA); Bruker Avance III 400 MHz nuclear magnetic resonance spectrometer (Bruker, Billerica, MA, USA); Thermo Nicolet iS50 Fourier-transform infrared spectrometer (Thermo Fisher Scientific, Waltham, MA, USA); Malvern Zetasizer Nano ZSP (Malvern Panalytical, Malvern, Worcestershire, UK); Anton Paar MCR 602 rotational rheometer (Anton Paar GmbH, Graz, Austria); Malvern Zetasizer Nano ZSP (Malvern Panalytical, Malvern, Worcestershire, UK) for particle size and Zeta potential measurement; Sigma500 scanning electron microscope (SEM; Carl Zeiss AG, Oberkochen, Germany); high-temperature and high-pressure flooding system; constant-temperature heating magnetic stirrer; DVIII-ultra rotational viscometer; Waring blender; electronic balance; and timer.

### 3.2. Experimental Methods

#### 3.2.1. Preparation of Partially Quaternized QPD with DMC

1. Synthesis of PDMAEMA Monomer Polymer

According to the molar ratio of DMAEMA monomer, chain transfer agent (DMP) and initiator (AIBN) at 2000:10:1, 31.4430 g of DMAEMA, 0.0364 g of DMP and 0.0016 g of AIBN were weighed and added into a reaction flask, followed by the addition of 32 mL of 1,4-dioxane (DO) as the solvent under stirring until complete dissolution. Nitrogen was purged into the solution for 30 min, and the flask was then sealed. The reaction was carried out under magnetic stirring at 75 °C; samples were taken periodically to monitor monomer conversion and molecular weight throughout the reaction. Upon completion, the mixture was cooled in an ice bath, diluted with DO, and precipitated with petroleum ether. The precipitate was further purified via repeated dissolution in DO and precipitation with petroleum ether for several cycles. The final product was vacuum-dried at 60 °C for 48 h to obtain purified PDMAEMA.

2. Preparation of DMC-Quaternized PDMAEMA

**Figure 16:**
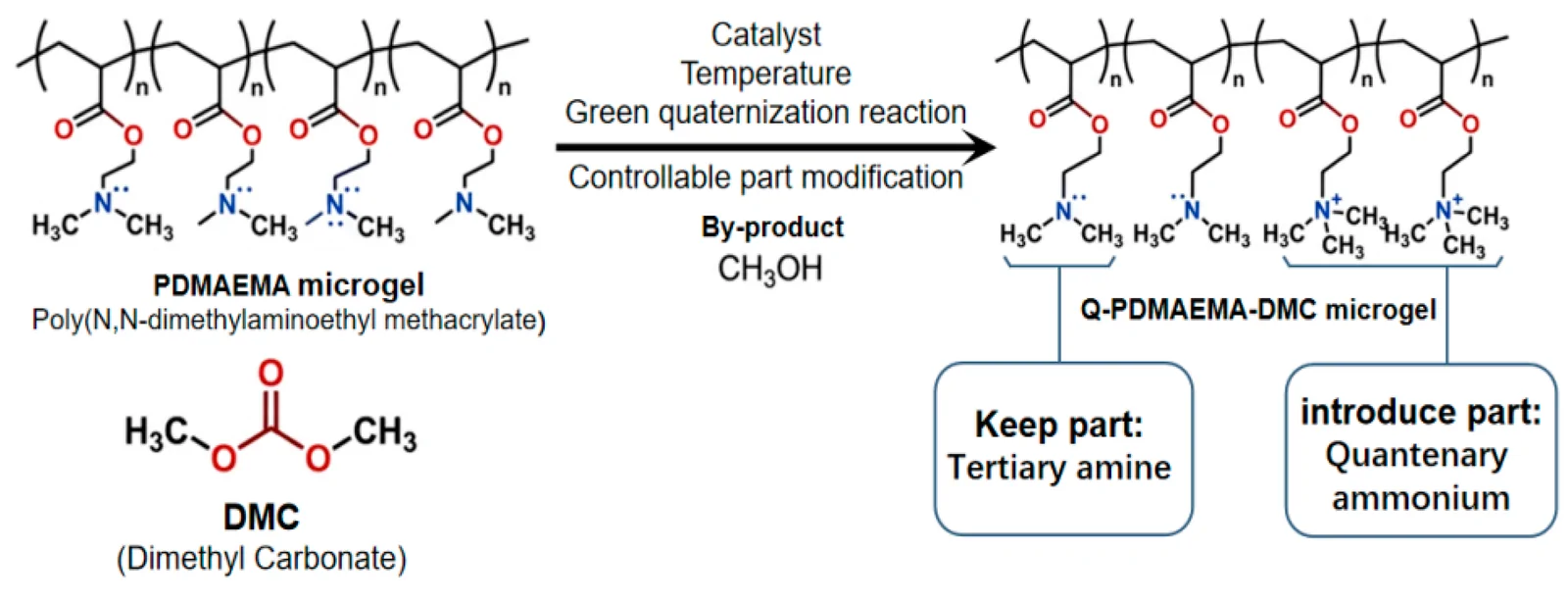
Synthetic route of the QPD gel.

Dissolve 1.0 g of PDMAEMA thoroughly in 30 mL anhydrous methanol. The reactants were dosed at a molar ratio of tertiary amine groups to DMC of 1:0.65, then placed in a high-pressure reactor for reaction at 120 °C over 7 h. After the reaction finished, the mixture was processed via rotary evaporation, dialysis and freeze-drying in sequence, yielding QPD as a white powdered solid. This synthesis route adopts green reagent DMC to replace conventional toxic alkyl halides. Meanwhile, the methanol produced as a by-product from DMC can be recycled and reused. Accordingly, an eco-friendly partial quaternization modification for the gel material was successfully achieved. The synthetic route for QPD gel is shown in Figure 16.

#### 3.2.2. Structural Characterization Tests

1. ^1^H Nuclear Magnetic Resonance (^1^H NMR) Testing

PDMAEMA and QPD samples were prepared at a concentration of 800 mg/L and dissolved in 0.6 mL of heavy water (D_2_O). After ultrasonic dispersion and static defoaming treatment, the tests were performed on a Bruker Avance III 400 MHz NMR spectrometer (Bruker, Billerica, MA, USA) at 25 °C with 128 cumulative scans to acquire the ^1^H NMR spectra. The results were then compared with those of the original PDMAEMA solution.

2. Fourier Transform Infrared Spectroscopy (FTIR) Analysis

A Thermo Nicolet iS50 infrared spectrometer (Thermo Fisher Scientific, Waltham, MA, USA) was employed to record the infrared absorption spectra of PDMAEMA and QPD. The scanning range was set from 4000 cm^−1^ to 400 cm^−1^ with a resolution of 4 cm^−1^. each sample was scanned 32 times in total to achieve a higher signal-to-noise ratio for clearer spectral results.

3. Microstructural analysis

The microstructures of the PDMAEMA and QPD gels were characterized using a Sigma500 scanning electron microscope (SEM; Carl Zeiss AG, Oberkochen, Germany). Samples were rapidly frozen in liquid nitrogen, followed by freeze-drying. Cross-sectional specimens (approximately2 mm × 2 mm × 2 mm) were cut from the center of the freeze-dried samples, mounted onto SEM stubs using conductive adhesive, and gently cleaned with a stream of nitrogen gas to remove surface debris. Subsequently, the specimens were sputter-coated with gold (15 mA current, 30 s duration, resulting in a coating thickness of approximately 10 nm). Imaging was performed at an accelerating voltage of 5 kV and a working distance of 8–10 mm. Each sample was examined three times.

4. Zeta Potential Measurement

PDMAEMA and QPD were separately dispersed in deuterated water to prepare 0.1 mg/mL gel solutions. All prepared solutions were treated with ultrasound for five minutes to eliminate particle aggregation. Subsequently, a Malvern Zetasizer Nano ZSP instrument (Malvern Panalytical, Malvern, Worcestershire, UK) was used to measure the Zeta potential values, followed by a comparative analysis against the data of pure PDMAEMA.

#### 3.2.3. Gelation Performance Testing

250 mL of simulated wastewater was prepared first. Precisely dosed amounts of 800 mg/L PDMAEMA and QPD, alongside 200 mg/L BS-99 stabilizer and 1800 mg/L YFJJ alkaline crosslinker, were added following the experimental formula. The mixture was stirred at 300 r/min for 2 h until all components dissolved completely. Once fully blended, the sample solutions were placed in a thermostatted water bath and held statically for 2 h at controlled temperatures of 40 °C, 50 °C, 60 °C, 83 °C and 100 °C, respectively. The apparent viscosity at the gelation point and the initial viscosity of the gel system at each temperature were measured using an Anton Paar MCR 602 rheometer (Anton Paar GmbH, Graz, Austria) at a fixed shear rate of 30 s^−1^. The apparent viscosity measured at the gelation point under a shear rate of 30 s^−1^ was defined as the gel strength.

#### 3.2.4. Rheological Performance Testing

Testing solutions were formulated by blending 250 mL of simulated reservoir water with 800 mg/L PDMAEMA or QPD, 200 mg/L BS-99 stabilizer, and 1800 mg/L YFJJ alkaline crosslinker. The mixture was stirred continuously at 300 r/min for two hours to ensure full dissolution of all components. Measurements were performed using an Anton Paar MCR 602 rheometer with a plate–plate geometry (rotor model: P35) at a test temperature of 83 °C. The plate gap was set to 1 mm and zero-calibrated to minimize errors. The viscosity curves of PDMAEMA and QPD gel solutions at various shear rates were measured, along with steady shear sweeps at 40 °C, 50 °C, 83 °C, 100 °C, and 120 °C (shear rate range: 0.1–1000 s^−1^). Stress sweep tests were conducted at a fixed frequency of 1.0 Hz over a stress range of 0.01–100 Pa to determine the linear viscoelastic region (LVR) and identify the appropriate stress value. Dynamic frequency sweep tests were then performed using the determined stress from the stress sweep, over a frequency range of 0.01–100 Hz. All rheological measurements were performed in triplicate (*n* = 3), and the curves presented in the figures represent the most representative data selected from the repeated measurements.

#### 3.2.5. Oil Flooding Performance Test

**Figure 17:**
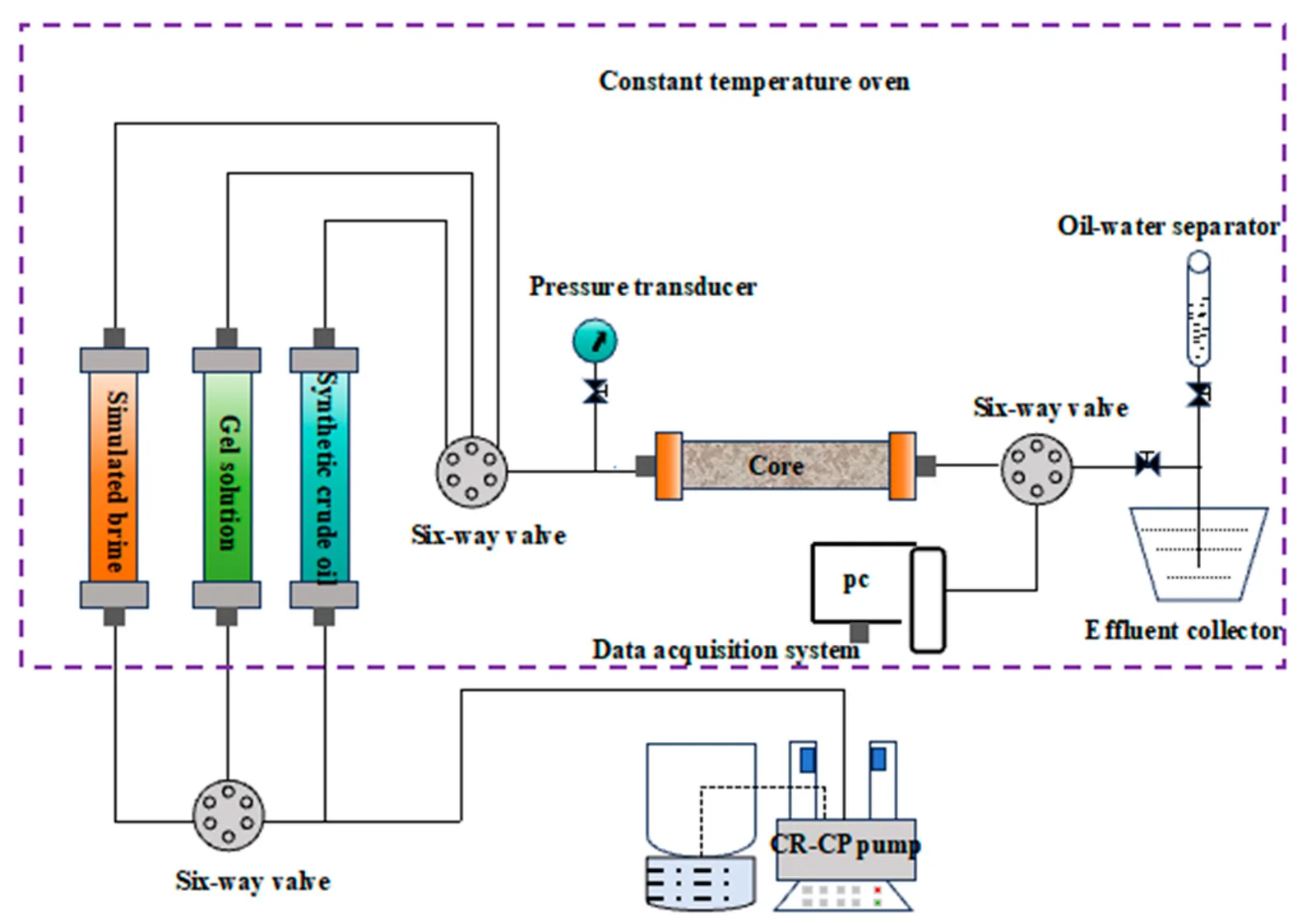
Experimental apparatus for oil displacement performance testing of three-layer heterogeneous rectangular cores.

This evaluation was designed to assess how effectively the PDMAEMA and QPD gel systems could enhance oil recovery under the simulated reservoir conditions of Block X. All experiments adopted artificial three-layer heterogeneous rectangular core samples, performed at 83 °C with a water salinity of 16,000 mg/L. The experimental setup is shown in Figure 17. The detailed testing workflow is outlined below:

Each core was vacuum-treated and fully saturated with water to measure its porosity and pore volume, then kept under constant temperature for no less than 12 h.

After water permeability measurement, the initial oil saturation was determined.

Water flooding was implemented at an injection rate of 0.3 mL/min, continuing until the water cut reached 96%. The recovery factor achieved by conventional water flooding was then calculated.

A tailored profile control system was injected at the same flow rate of 0.3 mL/min, with a total injection volume of 0.3 PV. The formulated system contained 800 mg/L PDMAEMA or QPD gel, 200 mg/L BS-99 stabilizer, and 1800 mg/L YFJJ alkaline crosslinking agent.

Subsequent water flooding was continued until the water cut rose to 98%, and the final oil recovery factor was calculated accordingly.

Real-time pressure data, water cut variations and oil recovery were recorded throughout every flooding stage, and the corresponding performance curves were plotted accordingly.

## 4. Conclusions

This study constructs a novel thermoresponsive gel system by partially quaternizing poly(N,N-dimethylaminoethyl methacrylate) (PDMAEMA) copolymers using green dimethyl carbonate (DMC) as an alternative to halogenated alkanes. By tuning the molar ratio of the tertiary amine to DMC to 1:0.65, a structure featuring 65% quaternization alongside 35% unreacted tertiary amine groups is achieved. This architecture retains the polymer’s multi-stimuli responsiveness while significantly enhancing cationic electrostatic repulsion and backbone rigidity through the introduced quaternary ammonium groups, thereby optimizing both molecular conformation and material performance.

Structural characterization (^1^HNMR, FTIR) confirmed successful quaternization modification. SEM observation revealed that the unmodified PDMAEMA gel, containing only tertiary amine groups and lacking strong electrostatic repulsion, relied on weak hydrophobic association and hydrogen bonding for physical crosslinking, resulting in a low crosslinking density and a loose network. After freeze-drying, it exhibited a macroporous three-dimensional structure with thin pore walls and good interconnectivity. In contrast, the introduction of quaternary ammonium groups in the modified QPD gel enhanced electrostatic interactions and hydrogen bonding, rendering the network denser and more stable, with fewer macropores, reduced pore size and thicker pore walls. These micromorphological differences are more pronounced than those reported for conventional HPAM-based gel systems which typically suffer from network collapse under high-temperature conditions. Zeta potential measurements showed that the surface potential of QPD increased from +20 mV to +40 mV, confirming successful exposure of quaternary ammonium groups. Compared with the partially quaternized PDMAEMA systems reported by Vamvakaki et al. and Manouras et al. using conventional alkyl halides, our DMC-based approach achieves a comparable level of surface charge enhancement while offering a greener and safer alternative. Gelation performance tests further demonstrated that the QPD gel system exhibits exceptional adaptability across a broad temperature range (40–120 °C) and superior gel strength. Unlike conventional phenolic gel systems, which exhibit poor gelling properties and insufficient stability under harsh reservoir conditions above 83 °C, the QPD gel maintained robust gel strength even at 100 °C. Rheological analysis confirmed substantial enhancements in the modified system’s three-dimensional network strength, zero-shear viscosity, thermosensitive response, and structural self-recovery capability. The QPD gel exhibited G′ > G″ across the tested frequency range with a low tan δ value, indicating stable solid-like elastic behavior—a significant improvement over the weak gel performance of unmodified PDMAEMA. Furthermore, the pronounced temperature–shear synergistic response of QPD demonstrates distinct advantages over conventional HPAM/chromium systems, which suffer from rapid backbone chain breakage and sharp viscosity loss at elevated temperatures.

Core flooding experiments were performed at 83 °C under a salinity of 16,000 mg/L. After injecting the gel at 0.3 PV, the QPD system reached an injection pressure of 1.4 MPa, noticeably higher than that of the original PDMAEMA gel. This proves its outstanding capacity for deep migration and strong in-depth plugging. During the subsequent water flooding stage, the water cut decreased rapidly, and the ultimate oil recovery reached 60%. This represented an improvement of 16.9 percentage points over conventional water flooding, and a further 6.2 percentage points compared with the PDMAEMA gel system. Due to poor temperature and salt resistance, the PDMAEMA gel showed low injection pressure and weak profile modification, achieving a final recovery of only 55%. Overall findings confirm that the partial quaternization strategy mediated by DMC can jointly enhance the gel’s thermal stability, salt tolerance and capacity for fluid diversion under harsh reservoir conditions.

Overall, these findings confirm that the DMC-mediated partial quaternization strategy effectively enhances the gel’s thermal stability, salt tolerance, and fluid diversion capacity under harsh reservoir conditions. Compared with traditional quaternization methods relying on toxic alkyl halides and conventional gel systems susceptible to thermal degradation, our approach achieves a synergistic improvement in environmental friendliness, structural stability, and oil recovery enhancement. Therefore, the DMC-mediated partial quaternization strategy offers a green and feasible approach for designing high-performance gel materials for profile control and flooding in high-temperature and high-salinity reservoirs.

## Data Availability

The data presented in this study are contained within the article.
